# A framework for the detection of *de novo* mutations in family-based sequencing data

**DOI:** 10.1038/ejhg.2016.147

**Published:** 2016-11-23

**Authors:** Laurent C Francioli, Mircea Cretu-Stancu, Kiran V Garimella, Menachem Fromer, Wigard P Kloosterman, Genome of the Netherlands consortium, Cisca Wijmenga, Principal Investigator, Morris A Swertz, Cornelia M van Duijn, Dorret I Boomsma, PEline Slagboom, Gertjan B van Ommen, Paul IW de Bakker, Morris A Swertz, Laurent C Francioli, Freerk van Dijk, Androniki Menelaou, Pieter BT Neerincx, Sara L Pulit, Patrick Deelen, Clara C Elbers, Pier Francesco Palamara, Itsik Pe'er, Abdel Abdellaoui, Wigard P Kloosterman, Mannis van Oven, Martijn Vermaat, Mingkun Li, Jeroen FJ Laros, Mark Stoneking, Peter de Knijff, Manfred Kayser, Jan H Veldink, Leonard H van den Berg, Heorhiy Byelas, Johan T den Dunnen, Martijn Dijkstra, Najaf Amin, K Joeri van der Velde, Jouke Jan Hottenga, Jessica van Setten, Elisabeth M van Leeuwen, Alexandros Kanterakis, Mathijs Kattenberg, Lennart C Karssen, Barbera DC van Schaik, Jan Bot, Isaäc J Nijman, Ivo Renkens, David van Enckevort, Hailiang Mei, Vyacheslav Koval, Karol Estrada, Carolina Medina-Gomez, Kai Ye, Eric-Wubbo Lameijer, Matthijs H Moed, Jayne Y Hehir-Kwa, Robert E Handsaker, Steven A McCarroll, Shamil R Sunyaev, Paz Polak, Dana Vuzman, Mashaal Sohail, Fereydoun Hormozdiari, Tobias Marschall, Alexander Schönhuth, Victor Guryev, Paul IW de Bakker, P Eline Slagboom, Marian B Beekman, Anton JM de Craen, H Eka D Suchiman, Albert Hofman, Cornelia M van Duijn, Ben Oostra, Aaron Isaacs, Najaf Amin, Fernando Rivadeneira, André G Uitterlinden, Dorret I Boomsma, Gonneke Willemsen, Mathieu Platteel, Steven J Pitts, Shobha Potluri, Purnima Sundar, David R Cox, Qibin Li, Yingrui Li, Yuanping Du, Ruoyan Chen, Hongzhi Cao, Ning Li, Sujie Cao, Jun Wang, Jasper A Bovenberg, Margreet Brandsma, Kaitlin E Samocha, Benjamin M Neale, Mark J Daly, Eric Banks, Mark A DePristo, Paul IW de Bakker

**Affiliations:** 1 Department of Medical Genetics, Center for Molecular Medicine, University Medical Center Utrecht, Utrecht, The Netherlands; 2 Analytic and Translational Genetics Unit, Massachusetts General Hospital, Boston, MA, USA; 3 Program in Medical and Population Genetics, The Broad Institute of Harvard and MIT, Cambridge, MA, USA; 4 Wellcome Trust Centre for Human Genetics, University of Oxford, Roosevelt Drive, Oxford, UK; 5 Department of Psychiatry, Icahn School of Medicine at Mount Sinai, New York, NY, USA; 6 Department of Genomic Sciences, Icahn School of Medicine at Mount Sinai, New York, NY, USA; 7 Department of Epidemiology, Julius Center for Health Sciences and Primary Care, University Medical Center Utrecht, Utrecht, CG, The Netherlands

## Abstract

Germline mutation detection from human DNA sequence data is challenging due to the rarity of such events relative to the intrinsic error rates of sequencing technologies and the uneven coverage across the genome. We developed PhaseByTransmission (PBT) to identify *de novo* single nucleotide variants and short insertions and deletions (indels) from sequence data collected in parent-offspring trios. We compute the joint probability of the data given the genotype likelihoods in the individual family members, the known familial relationships and a prior probability for the mutation rate. Candidate *de novo* mutations (DNMs) are reported along with their posterior probability, providing a systematic way to prioritize them for validation. Our tool is integrated in the Genome Analysis Toolkit and can be used together with the ReadBackedPhasing module to infer the parental origin of DNMs based on phase-informative reads. Using simulated data, we show that PBT outperforms existing tools, especially in low coverage data and on the X chromosome. We further show that PBT displays high validation rates on empirical parent-offspring sequencing data for whole-exome data from 104 trios and X-chromosome data from 249 parent-offspring families. Finally, we demonstrate an association between father's age at conception and the number of DNMs in female offspring's X chromosome, consistent with previous literature reports.

## Introduction

*De novo* mutation (DNM) between generations is a key mechanism in evolution. In humans, the mutation rate is estimated between 1 × 10^−8^ and 3 × 10^−8^ per base per generation from direct observations^1, 2, 3, 4^ and from species comparisons,^5^ although mutation rates have been shown to vary locally,^2, 6^ across families^2, 3, 4^ and to depend on paternal age.^3^ While most DNMs are thought to be selectively neutral, the phenotypic consequences can be severe when functional elements in the genome are mutated,^7^ and such cases are therefore of critical interest for medical genetics.^8^

Next generation sequencing (NGS) technologies applied to whole genomes in pedigrees enable systematic discovery and analysis of DNMs. Because the error rates from NGS data are currently much greater than the underlying DNM rate, detecting DNMs from NGS data requires accurate, quantitative calibration of the evidence supporting a novel allele in the offspring and the evidence against Mendelian transmission of this allele from (one of) the parents. A miscalled genotype in the parents or the offspring may lead to a false positive or false negative result. Consequently, variant callers^9, 10^ emit genotype likelihoods for each possible genotype to incorporate the uncertainty from the raw data.

We developed an algorithm called PhaseByTransmission (PBT) to compute the posterior probability for each genotype combination within a trio at each site given the genotype likelihoods in the individual family members, the known familial relationships and (optionally) the allele frequency in the population. PBT considers bi-allelic single nucleotide variants (SNVs) and short insertions and deletions (indels) within the autosomes and the X chromosome, and generates a list of all candidate DNMs ranked by their posterior probability. A key advantage is the integration of PBT within the widely used Genome Analysis Toolkit (GATK)^9^ and its ability to leverage phase information from the GATK ReadBackedPhasing module to identify the parental origin of DNMs.

## Materials and methods

PhaseByTransmission takes individual genotype likelihoods as input, defined as the likelihood *L* of the bases *D* observed at a site given each bi-allelic genotype *G*: *L*(*D*|*G*). These likelihoods can be computed from the sequence data using different genotype calling algorithms, such as the GATK UnifiedGenotyper (UG), GATK HaplotypeCaller or Samtools.^11^

For a given parent–parent–offspring trio, we enumerate all possible genotype combinations at a unique site in the genome. For bi-allelic autosomal sites, there are 27 possible genotype combinations within a trio: 15 are consistent with Mendelian inheritance, 10 correspond to a single DNM and 2 correspond to a pair of DNMs (involving a mutation from both parents). For bi-allelic sites on the X chromosome of a female offspring, only 18 genotype combinations exist because the father is haploid: 8 are consistent with Mendelian inheritance, 8 correspond to a single DNM and 2 correspond to a pair of DNMs. Because male offspring are haploid on the X chromosome and inherited their X chromosome from their mothers, there are only 6 mother-offspring genotype combinations: 4 are consistent with Mendelian inheritance and 2 correspond to a single DNM.

Given a mutation rate *μ*, *n* genotype combinations consistent with a single DNM (from 1 parent) and *m* genotype combinations consistent with two DNMs (from both parents), we define the following genotype combination prior:


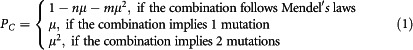


By using these genotype combination priors, we can compute the posterior probability of observing the sequencing data *D* given each of these possible underlying genotype combinations:


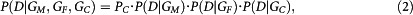


where *G*_*M*_, *G*_*F*_ and *G*_*C*_ are the genotypes of the mother, father and child, and *P*_*C*_ the genotype combination prior.

Following the posterior calculation for each of the *N* possible genotype combinations in the trio, we assign the most likely one to the trio, at each site, and compute its normalized posterior probability. All sites and trios assigned a genotype combination violating Mendel's laws are reported as putative DNMs and the posterior probability assigned to each of them reflects the confidence of the call. In addition to the familial relationships among samples, population allele frequencies can be incorporated as a prior into our model. Because one of the most common sources of false positive DNM calls is lack of sequence coverage in (one of) the parents, informing the model about allele frequencies in the population can help to reduce false positive rates. When adding allele frequency priors, Equation(2) becomes:


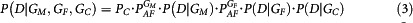


where *G*_*M*_, *G*_*F*_ and *G*_*C*_ are the genotypes of the mother, father and child, 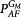 and 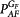 the allele frequency priors for the mother's and father's genotypes, and *P*_*C*_ the genotype combination prior.

The allele frequencies for the sites can be provided either as a separate VCF file or computed from the genotypes of the samples in the input VCF file when multiple samples from a single population are studied. In this case, the allele frequencies are estimated as 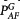 for each genotype *G* following Hardy-Weinberg equilibrium expectation:


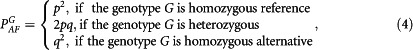


where *p* and *q* are the estimated allele dosage for the reference and alternate alleles, respectively, in the parents (founders).

In addition to calling DNMs, PBT also phases the inherited variants based on the segregation of alleles within a trio. By considering all possible genotype combinations and following Mendelian inheritance, we can infer phase deterministically for all trio individuals in all but two situations: when all trio individuals are heterozygous for the same two alleles, or when there is a DNM in the offspring. Except for these two cases, the phasing quality is bounded by the joint probability of the trio genotype combination.

## Results

### Simulated data

**Figure 1:**
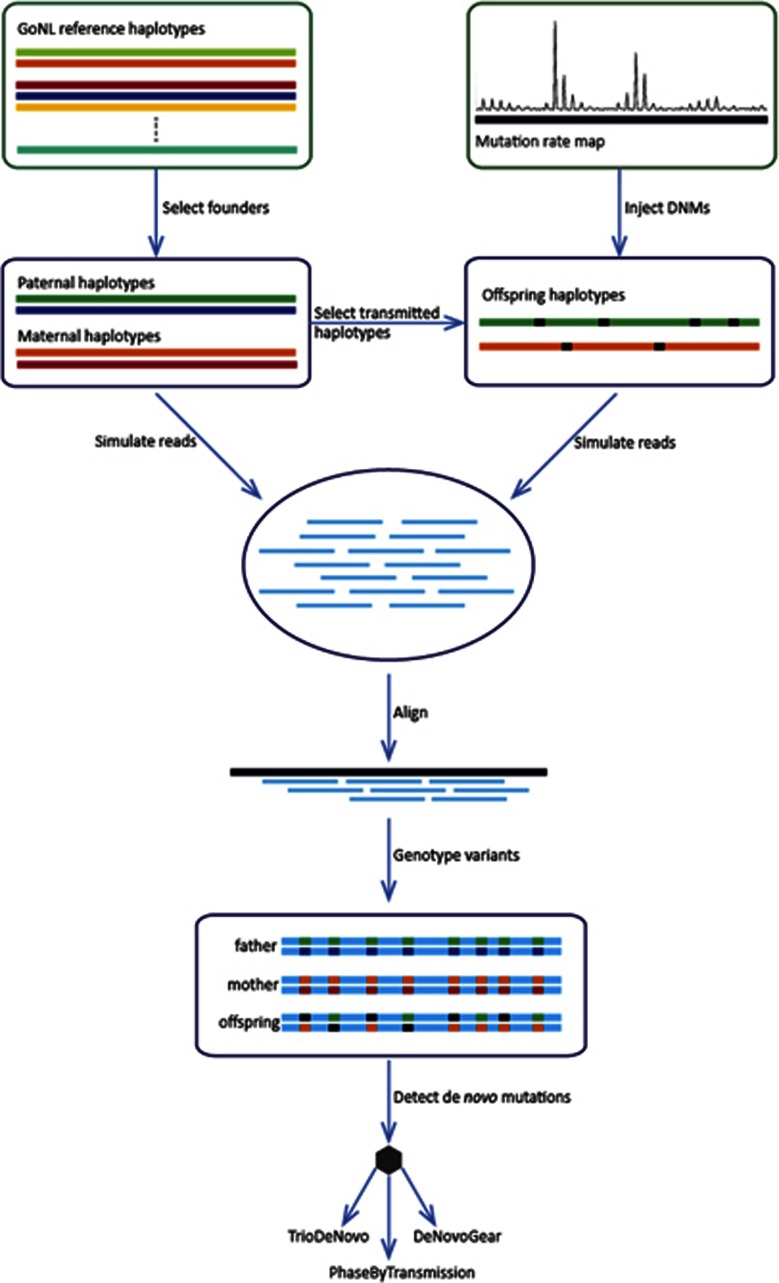
Outline of the pipeline used to generate our simulation data. The ‘mutation rate map' is the autosome-wide GoNL derived mutation rate map, as published before.^12^

In order to evaluate the performance of PBT we simulated sequencing data for 10 parent-offspring trios, 5 with a male offspring and 5 with a female offspring (Figure 1). We randomly selected 10 families from the Genome of the Netherlands (GoNL) Project^4^ and used previously reconstructed haplotypes for the parents for our simulations. We created haplotypes for the children by randomly selecting one haplotype from each of the parents and introduced on average 11 435 DNMs across the autosomes and 1821 on the X chromosome per offspring (all single base changes). In order to obtain a realistic genome-wide distribution of DNMs, we applied substitution-specific local mutation probabilities, which we empirically derived from the GoNL mutation rate map.^12^ This mutation map covers 75% of the human genome and provides mutation rate estimates at the megabase scale for all substitution types, as well as for C>T transitions in a CpG context. To simulate the paternal bias observed in previous studies,^2, 3, 4^ we randomly assigned 70% of the DNMs to the paternal haplotype and 30% of them to the maternal haplotype. Mutations across the X chromosome were distributed uniformly, as no mutation map was available. We used SimSeq^13^ to simulate 100 bp Illumina paired-end reads with an insert size of 250 bp for all 30 samples, within 10 kb regions centred on each simulated DNM (5 kb upstream and 5 kb downstream). We used the SimSeq default Illumina error profile in our simulation, which inserts errors (and their corresponding phred quality scores) in the simulated reads, as a function of the position within the read and the underlying reference base. The reads were aligned to the UCSC human reference sequence build 37 using BWA^14^ to produce aligned BAM files. To evaluate the effect of depth of coverage on DNM detection, we downsampled the generated BAM files for each sample during the variant calling step, to obtain variant call sets for average depths of coverage of 60x, 30x and 15x, respectively. The GATK UG was used on each trio separately to produce the individual genotype likelihoods used as input for PBT.

Using the UG default settings, an average 96% of the simulated DNMs were called as putative variant sites (the remaining 4% were not detected). The VCF file for each trio comprised, on average, 175 458 inherited SNVs and 11  427 Mendelian violations per trio. We ran PBT on the input VCF files using a mutation prior of 1.5 × 10^−8^ based on estimated per-base human mutation rate estimate.^1, 2, 3^ We also explored more permissive mutation priors (10^−7^, 10^−6^, 10^−5^ and 10^−4^) and assessed sensitivity and specificity of the downstream results as a function of the depth of coverage and mutation prior. In addition, we ran PBT with and without allele frequency priors based on 1000 Genomes Phase 3 CEU data.^15^ We ran PBT on each set of parameters, and computed the following: the number of simulated DNMs reported as DNMs by PBT (true positives); the number of inherited variants and sequencing errors reported as DNMs by PBT (false positives); the number of inherited variants not reported as DNMs by PBT (true negatives); the number of simulated DNMs not reported as DNMs by PBT (false negatives). From these, we computed the sensitivity as 
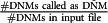
 and the specificity as 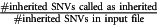.

**Figure 2:**
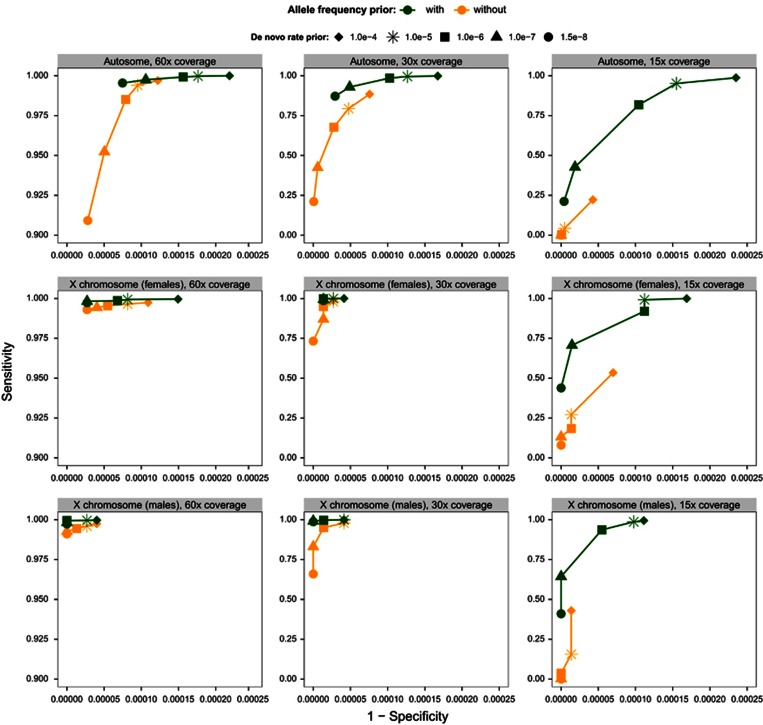
ROC plot showing the performance of PBT, where the mutation rate prior is used as the hidden parameter. Two scenarios are considered in order to evaluate the relevance of using allele frequency priors (yellow curve: without AF priors, green curve: with AF prior). The analysis is stratified by coverage (columns) and genomic region (rows). The y-scale for the 60x coverage plots is restricted for visibility. Each dot shape corresponds to a specific DNM prior. The allele frequency priors are computed based on 1000 Genomes Phase 3 CEU data.

Figure 2 shows the influence of the mutation rate prior and the allele frequency prior on the receiving operator characteristic (ROC) curves for both autosomes and the X chromosome at different depths of coverage. The mutation prior affects the sensitivity and specificity of the resulting DNM calls. As expected, a higher mutation prior increases the sensitivity at the cost of more false positive calls. As a result, the mutation prior value needs to be set depending on the desired output and the sequencing coverage (Figure 2). We note that as coverage increases the optimal value for real data should converge towards the actual human mutation rate (as can be seen for the 60x coverage data). Incorporating allele frequency priors into DNM detection greatly improved the sensitivity at low and medium coverage for both autosomes and the X chromosome. This reflects the higher uncertainty of the parents' genotypes at lower coverage, resulting in poor discrimination between homozygous and heterozygous genotypes. Incorporating the allele frequencies in the model thus leads to a better discrimination between (a) a site that is variant in the population and thus likely to be inherited from one of the parents even though there is little (or no) evidence for the variant allele in (one of) the parents, and (b) a site that is not variant in the population and is likely to be *de novo* if there is no evidence for the variant allele in either parents.

**Figure 3:**
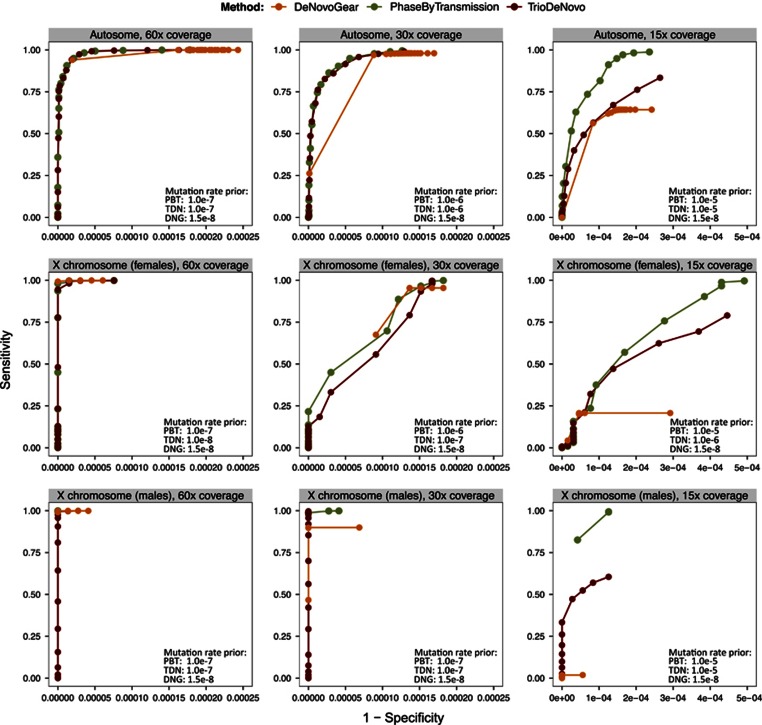
ROC plot illustrating the performance of three DNM calling methods (PhaseByTransmission, TrioDeNovo and DeNovoGear), with respect to each method's DNM output confidence score. The analysis is stratified by coverage (columns) and genomic region (rows). The posterior cutoffs used for plotting each curve were uniformly distributed across the range of each tool's output DNM confidence scores. Some outlier values where the specificity decreased considerably without any sensitivity gain were removed from the plot and the x-scale for the 60x and 30x coverages is restricted, for visibility purpose. Supplementary Figure SF3 shows the curves with all points. The mutation rate prior values for each scenario, for each tool are selected based on Supplementary Figure SF1.

To compare the performance of PBT against other state-of-the-art DNM callers, we used the same input VCFs to detect DNMs with TrioDeNovo^16^ and DeNovoGear.^17^ We selected these DNM callers, for their good reported performance as well as similar integration points within analysis pipelines (ie, after individual variant calling is performed). We used the best performing DNM rate prior (out of five predefined priors: 1.5 × 10^−8^, 10^−7^, 10^−6^, 10^−5^ and 10^−4^) to obtain DNM call sets, for each method and coverage. For PBT, we used the allele frequency prior as well. The optimal (in terms of sensitivity versus specificity) mutation rate prior's values were derived from Figure 2 for PBT and from a similar analysis (ie, influence of the mutation rate prior on specificity and sensitivity), on the same simulated dataset, for TrioDeNovo and DeNovoGear (Supplementary Figures SF1 and SF2). The mutation rate parameter value for each method is consistent with documentation or recommendations for each of the tools, where available. Figure 3 shows the ROC curves for the autosomes and the X chromosome at different depths of coverage using the posterior probability reported by each tool as parameter.

All three tools surveyed in this analysis performed very well in terms of the sensitivity at high coverage, while PBT and TrioDeNovo exhibit slightly better specificity. At lower coverage, the differences in sensitivity and specificity become more pronounced. The performance gain achieved by PBT at lower coverage comes from the incorporation of the allele frequencies in the model, which allows for a better discrimination between poorly covered variant sites in the parents and true DNMs. PBT showed good performance in detecting DNMs on the X chromosome even at lower coverage (15x), which was particularly challenging for the other two methods, especially in the male offspring trios. PBT had a sensitivity of 99% in female offspring trios and 98% in male offspring trios. In contrast, TrioDeNovo detects only 77% and 58% of female and male offspring DNMs on the X chromosome respectively, and DeNovoGear sensitivity drops down to 24% for the female offspring DNMs and 3% for the male offspring DNMs, respectively. The better performance of PBT on the X chromosome comes from explicitly modelling the unique mode of inheritance for this chromosome, whereas other tools do not differentiate between autosomes and the X chromosome.

We further evaluated our ability to assign parental origin to the DNMs identified. Assuming sequence reads are of sufficient length, heterozygous variants located close to the DNM can be informative about its parental origin and phase. To this end, we combined trio-based phasing information from PBT and read-based phasing information from ReadBackedPhasing in order to reconstruct the two haplotypes transmitted to the offspring. We only assigned parental origin to sites where all read data spanning adjacent offspring heterozygous positions unambiguously supported the same parental haplotype. We were able to determine parental origin for 14.1% of the simulated DNMs and 81.4% of these were assigned correctly. We note that other tools do not provide automated annotation of the parental origin.

### Empirical whole-genome data

In previous work, we have demonstrated the performance of PBT to detect *de novo* SNVs and indels in 13x coverage autosomal sequencing data of 250 parent-offspring families and on three parent-offspring families with both whole-exome and whole-genome data from the CLARITY challenge.^18^

Here, we present the application of PBT on the X chromosome sequencing data of 249 parent-offspring families from the GoNL project (230 trios, 11 parent-offspring families with a pair of monozygotic twins and eight parent-offspring families with a pair of dizygotic twins). We used only one randomly chosen offspring from each family with monozygotic twins and used both offspring from families with dizygotic twins. This resulted in a total of 257 offspring (111 males, 146 females) for DNM calling. All GoNL samples were selected without phenotypic ascertainment so as to be representative of the general Dutch population. The DNA samples were extracted from whole blood, and sequenced on Illumina HiSeq2000 using 90 bp paired-end reads with an insert size of 500 bp. The reads were aligned to the UCSC human reference sequence build 37 using BWA and processed using GATK best practices (https://www.broadinstitute.org/gatk/guide/best-practices). SNVs were called using GATK UG and subsequently filtered using GATK VariantQualityScoreRecalibration (VQSR). We excluded the pseudo-autosomal regions from this analysis since the homology between the X and Y chromosomes in these regions causes ambiguous read mapping and unreliable subsequent genotype calls with current analysis pipelines. The resulting set comprised 701 910 SNVs on the X chromosome and a total of 872 214 Mendelian violations.

We applied PBT to these data using a mutation prior of 10^−5^, which should provide optimal sensitivity based on our simulations (Figure 2). We also used an allele frequency prior based on the observed allele frequency in all unrelated samples in our study. We applied a posterior cutoff of Q5 for female offspring and kept all DNM calls in male offspring regardless of their posterior, since male offspring calls had lower posteriors in general, due to their overall lower genotype quality. Using these permissive parameters and thresholds, PBT reported a total of 10 380 DNMs. Due to the low depth of sequencing in our data, many of the lower quality calls are likely to be false positives and we thus filtered this set by removing any DNM candidates with any read evidence for the non-reference allele in either of the parents (which in our sequencing context most likely indicates insufficient sequencing of the alternative allele). This resulted in a final set of putative DNMs of 126 male offspring DNMs and 547 female offspring DNMs.

We selected six putative DNMs in male offspring and 54 in female offspring for validation. These candidates were selected randomly from the 66 families where DNA was available for validation using MiSeq deep sequencing (~1200x coverage). The six male offspring DNMs originate from six different families, whereas the 54 female offspring DNMs originate from 15 families with a median of 3 DNMs per child and a maximum of 7. From the six candidates in male offspring, four could be successfully assayed and all were validated as a true DNM in the offspring. From 54 candidates in female offspring, 43 could be successfully assayed of which 42 (97.7%) were validated as a true DNM. For 10 of the 13 unsuccessfully assayed DNMs, the capture and/or amplification of the locus surrounding the DNM failed for at least one of the individuals in the trio. In the remaining three cases, the coverage produced by the sequencing run was low in all trio individuals (2–20x). In these three cases, the low coverage data was compatible with a DNM (alternate allele present in child only), but we did not consider the evidence to be sufficient to unambiguously validate the mutation as *de novo*.

**Figure 4:**
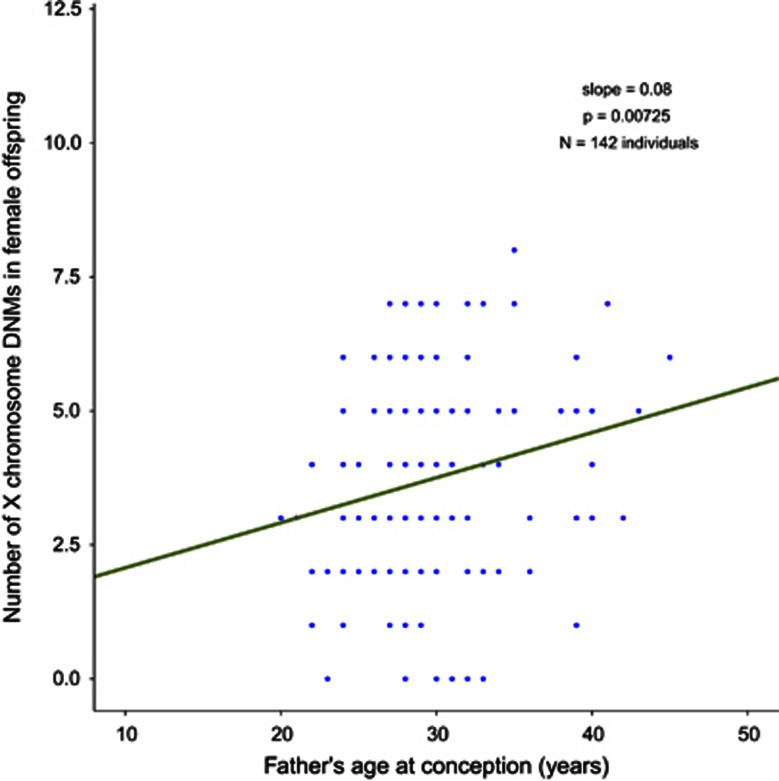
Fitted linear regression line (dark green) of the number of X chromosome DNMs, as a function of father's age at conception. The data points (blue) represent the set of 547 high confidence DNMs in female offspring. The coefficient estimate is an increase of 0.08 DNMs per year (on the X chromosome).

We found that male offspring carried on average 1.14 DNMs on the X chromosome, while female offspring carried 1.85 per copy of the X chromosome. Given that male offspring always inherit their X chromosome from their mothers, the much lower average number of DNMs found on the X chromosome of male offspring (1.14), when compared to female offspring (1.85 per copy), is compatible with the paternal germline being highly enriched for DNMs.^1^ Despite the limited number of observations in the study, we found a statistically significant increase of DNMs on chromosome X with paternal age in female offspring by fitting a linear regression model (*P*=0.00725), consistent with previous reports^2, 3, 4^ (Figure 4). As expected, this effect was absent in the male offspring (*P*=0.24). The linear estimate of 0.08 additional DNMs per year of paternal age on the X chromosome in female offspring data is consistent with previously obtained estimates based on autosomal DNMs (accounting for chromosome sequence length).

### Empirical whole-exome data

We evaluated our software on whole exome data in a cohort of 104 trios (single proband and parents). DNA was extracted from whole-blood and exons captured using the Agilent 38 Mb SureSelect v2 and sequenced at 60x average depth on the Illumina HiSeq2000 platform for an independent autism study.^19^ The sequence data were aligned to the human reference hg19 using BWA,^14^ duplicate reads removed, re-alignment performed around insertions/deletions, and base quality scores recalibrated. Variant discovery and genotyping was performed using the GATK UG across all samples jointly, and calls were subsequently filtered using GATK VQSR.^10^

We ran PBT with a mutation prior of 10^−7^, on the basis of our simulations (Figure 2), and an allele frequency prior based on the observed data (208 parents). In total, we called 148 putative DNMs, all of which were subjected to experimental validation using Sequenom, and 115 (77.8%) could be assayed successfully. From these, 107 (93%) candidates were validated as true DNMs in the offspring. Looking at false positive calls, five (4.7%) were monomorphic in all samples and three (2.8%) were inherited variants.

## Discussion

PhaseByTransmission is an efficient and automated DNM caller using a Bayesian model to estimate the probability of *de novo* SNVs and/or indel at each site in one or more trios. The model should in principle work with structural variants if genotype likelihoods can be provided. Because PBT works with VCF files as input, it can be integrated into existing NGS analysis pipelines and its results can be annotated using most impact-prediction tools. The PBT algorithm scales linearly with the number of sites and trios. Results on real sequencing data show excellent specificity and sensitivity at both lower and higher coverage in whole-exome and whole-genome data sets. Because PBT explicitly models the inheritance pattern for the X chromosome, it can also be used to derive accurate calls on the X chromosome of both male and female offspring. In addition, due to its integration with the GATK ReadBackedPhasing module, it can provide parent-of-origin information. Finally, PBT can also be used to infer the haplotype phase for most inherited variants in a trio based on the allele segregation within the trio.

### Availability of data and materials

PhaseByTransmission and ReadBackedPhasing are available as part of the GATK as a precompiled Java package as well as source code at http://www.broadinstitute.org/gatk/download. The GoNL data can be accessed at http://www.nlgenome.nl.
