# Supplementary material for: A framework for the detection of de novo mutations in family-based sequencing data
Source: Eur J Hum Genet. 2016 Nov 23;25(2):227–33. doi: 10.1038/ejhg.2016.147 (PMC5255947; doi:10.1038/ejhg.2016.147)
Supplement: Supplementary Figure 1 [file ejhg2016147x1.pdf]

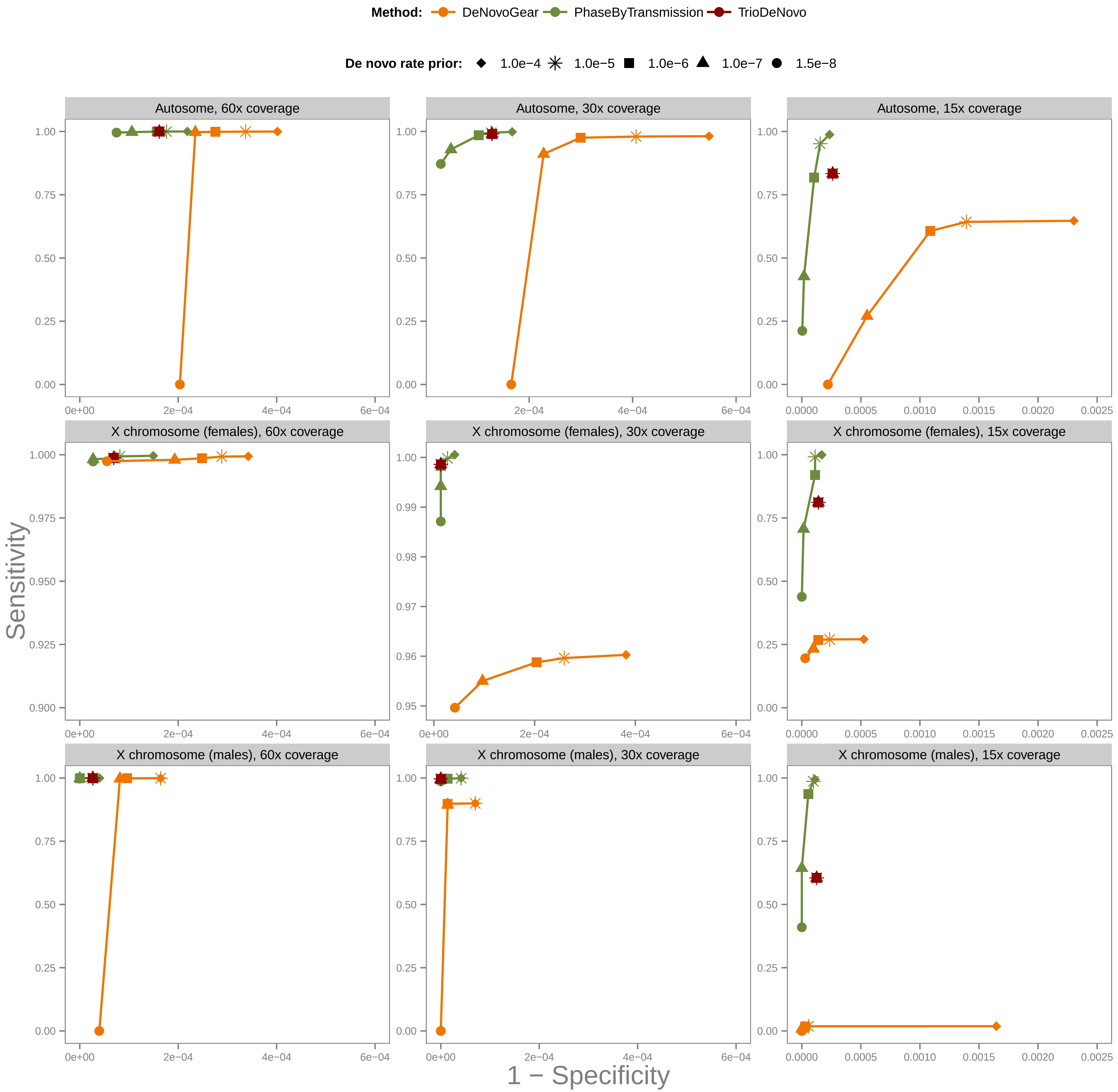

Method: DeNovoGear PhaseByTransmission TrioDeNovo
De novo rate prior: 1.0e−4 1.0e−5 1.0e−6 1.0e−7 1.5e−8
Autosome, 60x coverage
Autosome, 30x coverage
Autosome, 15x coverage
X chromosome (females), 60x coverage
X chromosome (females), 30x coverage
X chromosome (females), 15x coverage
X chromosome (males), 60x coverage
X chromosome (males), 30x coverage
X chromosome (males), 15x coverage
Sensitivity
1 − Specificity
0e+00 2e−04 4e−04 6e−04
0.0000 0.0005 0.0010 0.0015 0.0020 0.0025
0.00 0.25 0.50 0.75 1.00
0.900 0.925 0.950 0.975 1.000
0.95 0.96 0.97 0.98 0.99 1.00
