# Supplementary material for: A framework for the detection of de novo mutations in family-based sequencing data
Source: Eur J Hum Genet. 2016 Nov 23;25(2):227–33. doi: 10.1038/ejhg.2016.147 (PMC5255947; doi:10.1038/ejhg.2016.147)
Supplement: Supplementary Figure 2 [file ejhg2016147x2.pdf]

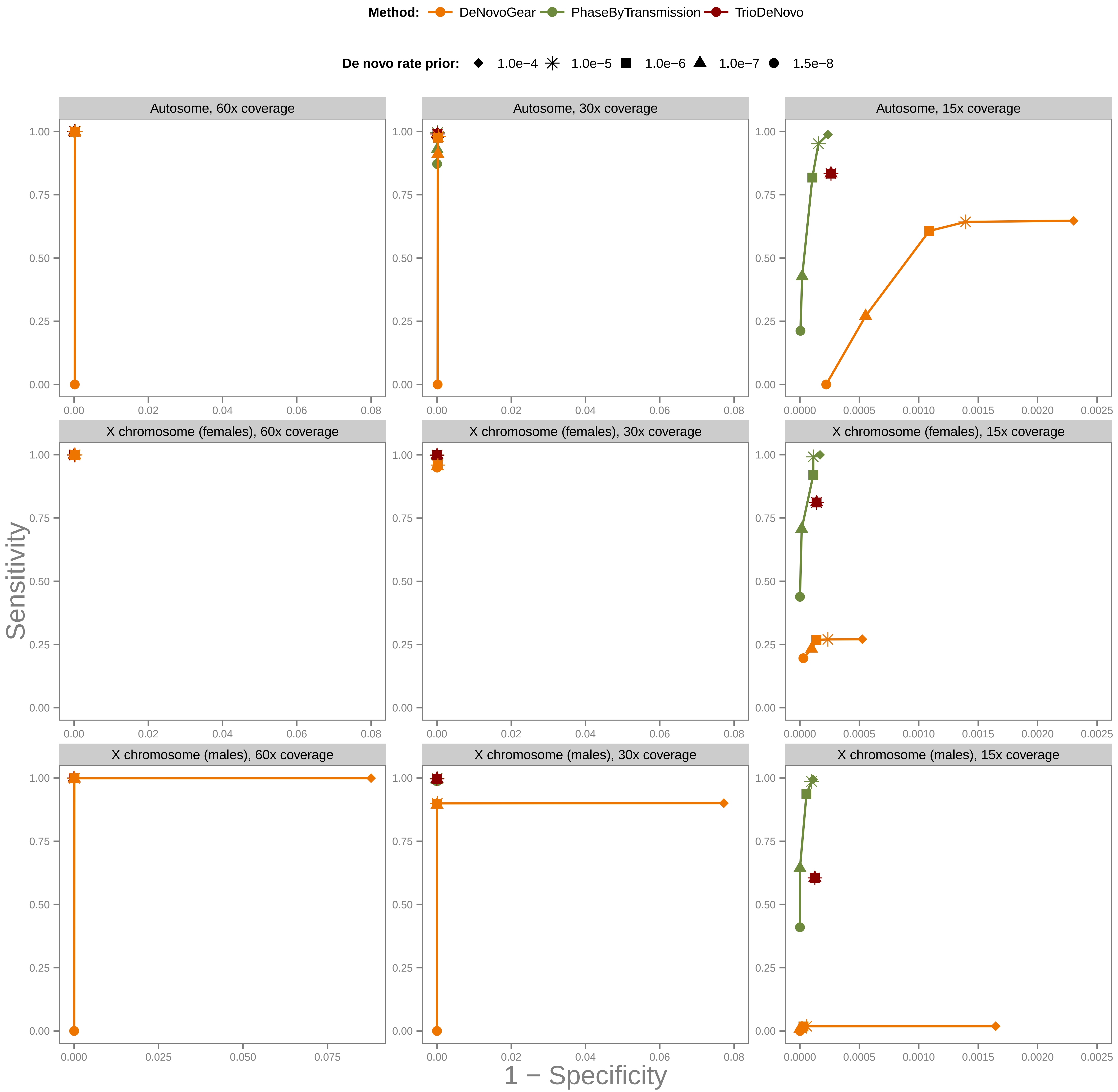
Method: DeNovoGear PhaseByTransmission TrioDeNovo
De novo rate prior: 1.0e−4 1.0e−5 1.0e−6 1.0e−7 1.5e−8
Autosome, 60x coverage
Autosome, 30x coverage
Autosome, 15x coverage
X chromosome (females), 60x coverage
X chromosome (females), 30x coverage
X chromosome (females), 15x coverage
X chromosome (males), 60x coverage
X chromosome (males), 30x coverage
X chromosome (males), 15x coverage
Sensitivity
1 − Specificity
0.00
0.25
0.50
0.75
1.00
0.02
0.04
0.06
0.08
0.0000
0.0005
0.0010
0.0015
0.0020
0.0025
0.000
0.025
0.050
0.075
