# Supplementary figures and images for: A framework for the detection of de novo mutations in family-based sequencing data
Source: Eur J Hum Genet. 2016 Nov 23;25(2):227–33. doi: 10.1038/ejhg.2016.147 (PMC5255947; doi:10.1038/ejhg.2016.147)

Method: DeNovoGear PhaseByTransmission TrioDeNovo

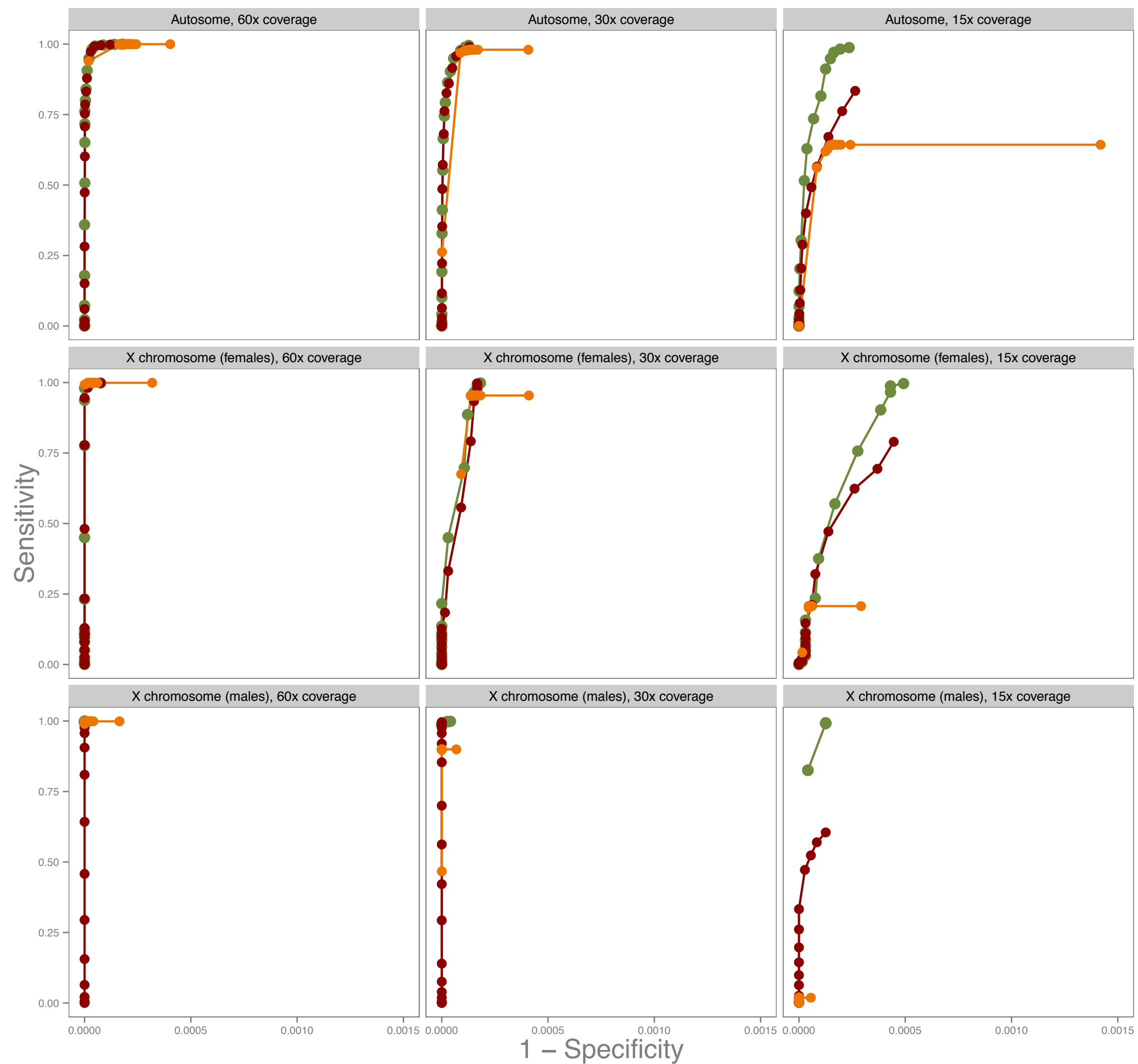

Supplement: Supplementary Figure 3 [file ejhg2016147x3.pdf]
